# Conservation Thinning in Secondary Forest: Negative but Mild Effect on Land Molluscs in Closed-Canopy Mixed Oak Forest in Sweden

**DOI:** 10.1371/journal.pone.0120085

**Published:** 2015-03-24

**Authors:** Birte Rancka, Ted von Proschwitz, Kristoffer Hylander, Frank Götmark

**Affiliations:** 1 Department of Biological and Environmental Sciences, University of Gothenburg, Göteborg, Sweden; 2 Museum of Natural History, Göteborg, Sweden; 3 Department of Ecology, Environment and Plant Sciences, Stockholm University, Stockholm, Sweden; Roehampton university, UNITED KINGDOM

## Abstract

Secondary succession is changing the character of many temperate forests and often leads to closed-canopy stands. In such forests set aside for conservation, habitat management alternatives need to be tested experimentally, but this is rarely done. The Swedish Oak Project compares two often debated alternatives: minimal intervention and non-traditional active management (conservation thinning) on plots of each type replicated at 25 sites. We study responses of several taxa, and here report results for land molluscs. They are considered to be sensitive to more open, drier forest and we predicted a negative effect of the thinning (26% reduction of the basal area; mean value for 25 experimental forests). We sampled molluscs in the litter in ten 20 x 25 cm subplots, and by standardised visual search, in each plot. In total, we recorded 53 species of snails and slugs (24 369 individuals) and the mean species richness in plots was 17. Two seasons after thinning, mean (± SE) species richness had decreased by 1.4 (± 0.9) species in thinning plots, but increased by 0.7 (± 1.0) species in minimal intervention plots, a significant but small change with considerable variation among sites. In matched comparisons with minimal intervention, thinning reduced the overall abundance of molluscs. Most species responded negatively to thinning – but only five of the 53 species were significantly affected, and reproduction seemed to be negatively affected in only one species. An ordination analysis did not reveal any particular change in the species community due to thinning. Thus, the negative effect of conservation thinning on land molluscs was apparently mild – one reason was that many trees, shrubs and other forest structures remained after the treatment. Conservation thinning may be recommended, since other taxa are favoured, but minimal intervention is also a useful form of management for molluscs and saproxylic taxa.

## Introduction

The area of protected forest is steadily growing [1,2]. With new goals established in the Convention on Biological Diversity in Nagoya [3], within 10–20 years some 15–20% of the temperate forest might be protected in various ways to secure biodiversity. Many of these forests are not old-growth (i.e., several hundred years old) and some taxa or species may be disfavoured by secondary succession and closed-canopy forest in formerly semi-open forests and woodland [4,5]. In this situation, agencies and managers of conservation forests sometimes will face difficult choices in habitat management. A recent review [6] outlined four management alternatives for forests where conservation is the main goal: minimal intervention, traditional management (e.g. woodland pasture), non-traditional management (e.g. thinning), and species management (e.g. for threatened species). Much research is needed to evaluate these alternatives, in particular critical field experiments [6].

In the Swedish Oak Project, which started in 2000, we compare and evaluate minimal intervention and non-traditional management in a long-term experiment (for an overview of the project, see 6). Using a BACI design (Before-After-Control-Impact) with 25 independent replicates (forest sites) at the regional level, we test hypotheses regarding the responses of taxa to the different management options. Our sites contain large oaks (*Quercus robur/Q*. *petraea*) and some other taxa that may need active management. The study plots are remnants of a former traditional land use that included widespread grazing across the landscape; after this land use was abandoned, long-term succession produced mixed, closed-canopy tall forests. By partial cutting, here referred to as conservation thinning or just ‘thinning’, we provide light and openings for some species that may then be favoured (e.g. *Quercus*, herbs, some saproxylic species). However, other species may be favoured by minimal intervention and succession towards old-growth. After our initial cutting in 2002/2003 (see below), only minor intervention actions are planned in thinning plots (e.g. to favour old oak trees, or oak regeneration).

We study seven taxa in the project; vascular plants, mosses, lichens, fungi, land molluscs, fungus gnats, and beetles, see e.g. [7–9]. In the present study, we investigated the short-term response of molluscs (snails and slugs) to conservation thinning and minimal intervention. The land molluscs are relatively species-rich in southern Sweden (about 130 species) and occur in high abundance on the forest floor in the broadleaved temperate forests. They mainly live in the litter, but also use stems and foliage and they are important for decomposition, as food for birds and other predators, and for other ecological functions [10–12]. Due to their small body sizes and restricted mobility [13,14], occurrence in special microhabitats [15,16] and requirements for moisture and protection [17], they are considered sensitive to disturbances such as logging, drainage and acidification [18,13]. In Sweden and elsewhere, molluscs are therefore used in monitoring work [17–20]. Effective monitoring, and measures motivated by monitoring results, require good empirical knowledge about the responses of taxa to various measures and management. Strong experimental field studies can provide information on cause and effect [6,21], and can guide decisions. For boreal forests, such studies exist [22,23] but we are not aware of similar work in temperate forest.

Here we report short-term results from conservation thinning in our mixed closed-canopy oak-rich forests, with respect to the response of the land mollusc fauna. Our general hypothesis was that this taxon would be negatively affected; we predicted that species richness should decline following thinning, and that possibly community composition would be affected. In addition, we examined the responses of individual species and also attempted to analyse reproduction among the molluscs after thinning. Our study confirmed the hypothesis, though the negative effect was mild, and probably short-term.

## Material and Methods

### Study area and plot characteristics

**Fig 1:**
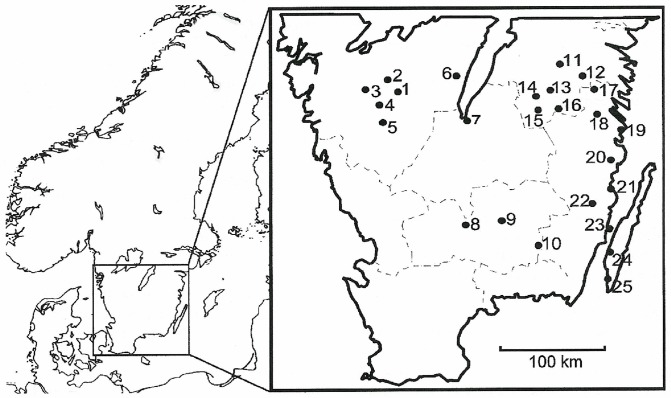
Study area in southern Sweden with the 25 forest sites used in the experiment. Each site contained one experimental thinning plot (1 ha) and one minimal intervention plot (1 ha), before the treatment similar in forest structure. For names and description of sites, see Table 1.

The 25 study sites are located in southern Sweden (Fig. 1), south of the boreal forest. Coniferous forest dominates the region and study area: production stands of *Picea abies* (L.) H. Karst and *Pinus sylvestris* L., with a minor component of birch *Betula* spp. (Swedish Statistical Yearbook of Forestry, on www.skogsstyrelsen.se). Mixed broadleaved forests of higher value for biodiversity form less than 10% of the total productive forest area in southern Sweden; 6.1% is protected or voluntary set-asides (‘Skogsstatistisk Årsbok’ in English, www.skogsstyrelsen.se)

Historically, much of the land was a mixture of woodland pasture, open pasture, and fields where oaks, *Quercus robur* L. and *Q*. *petraea* (Matt.) Liebl., were relatively common. During the succession that followed the abandonment of grazing (mainly by cattle) on large parts of the land, oaks spread to some extent, as did many other trees and shrubs (between about 1850–2000). In the Swedish Oak Project, we study 25 closed-canopy mixedwoods with large oaks (about 80–200 years old) at sites where agriculture ended 50–80 years ago. In the study area (Fig. 1), mean precipitation for July decreases from 80 mm at the western sites to 55 mm at the eastern coastal sites; variation among months is small (Swedish Meteorological and Hydrological Institute; www.smhi.se). The mean temperature in July varies from 14°C in the west to 17°C in the east; in January the mean temperature is just below 0°C (for more data, see www.smhi.se). The study sites are located 5–230 m above sea level.

We selected forests rich in large oaks due to the high conservation values associated with these trees [24,25] and the need for experimental habitat management. Our 25 study sites are nature reserves or part of the Forest Agency’s woodland key habitat network [26]. The sites have mesic moraine (till) and relatively level, partly stony ground. Brown earth soils dominate, with some podsol at four sites with much *Picea abies* (> 30% of basal area). A large clay fraction (10%) in the soil was recorded only at one coastal site, Vickleby (Fig. 1, Table 1).

**Table 1:** Characteristics of the 25 study sites on the map in Fig. 1.

Site	Basal area, trees (m^2^ / ha) > 5 cm diameter dbh			Other broadleaves, species^b^
	Conifers^a^	Oaks	Other	
1. Skölvene	1.2	18.2	9.0	*Alnus glutinosa*, *Betula spp*.
2. Karla	0.3	13.1	6.7	*Betula spp*., *Populus tremula*
3. Östadkulle	1.6	15.1	14.4	*Fraxinus excelsior*, *Populus tremula*
4. Sandviksås	15.1	10.2	5.5	*Betula spp*.
5. Rya åsar	1.3	22.8	7.9	*Betula spp*., *Sorbus aucuparia*
6. Strakaskogen	5.5	4.2	19.9	*Betula spp*., *Populus tremula*, *Salix caprea*, *Fraxinus excelsior*
7. Bondberget^c^	0.8	14.9	9.4	*Betula spp*., *Corylus avellana*
8. Långhult	0.2	11.8	15.8	*Tilia cordata*, *Betula spp*.
9. Bokhultet	5.7	16.2	12.3	*Fagus sylvatica*
10. Kråksjö by	2.3	20.3	13.3	*Betula spp*.
11. Stafsäter	0.0	6.0	15.6	*Tilia cordata*, *Fraxinus excelsior*
12. Åtvidaberg	4.5	17.2	16.5	*P*. *tremula*, *Betula spp*., *C*. *avellana*
13. Fagerhult	3.5	7.2	16.1	*Populus tremula*, *Corylus avellana*
14. Aspenäs	2.6	14.0	13.0	*Betula spp*.
15. Norra Vi	4.9	18.8	5.9	*Populus tremula*
16. Fröåsa	11.0	15.8	7.5	*Populus tremula*
17. Ulvsdal	1.4	10.1	13.0	*Tilia cordata*, *Populus tremula*
18. Hallingeberg	0.5	18.3	7.1	*Acer platanoides*
19. Ytterhult	3.5	14.4	5.5	—
20. Fårbo	8.7	17.1	5.4	—
21. Emsfors	0.3	10.6	15.8	*T*. *cordata*, *F*.*excelsior*, *A*. *platanoides*
22. Getebro	10.2	16.8	7.1	*Populus tremula*
23. Lindö	2.7	8.5	10.4	*Corylus avellana*, *Betula spp*.
24. Vickleby	0.0	26.5	4.3	*Corylus avellana*
25. Albrunna	0.0	11.9	17.2	*Fraxinus excelsior*
**Mean**	**3.5**	**14.3**	**10.6**	
**S.D.**	**4.0**	**5.3**	**5.1**	

^a^Overall 80% Picea abies, 20% Pinus sylvestris; only site 9 had > 10% Pinus of total basal area.

^b^Tree species with > 10% of total basal area given in decreasing percentage order

^c^Basal areas for Bondberget given earlier [37] are incorrect.

In each forest we delimited two plots in 2000 (each 1 ha, at most sites 100 m x 100 m). One plot was randomly assigned as treatment plot (conservation thinning) and the other as minimal intervention plot. To document the composition of trees before partial cutting, we measured basal area (stems > 5 cm in diameter at breast height) along transects covering 30–60% of each plot. The mean basal area per hectare and plot was 28 m^2^ (SD = 4.1). The dominant canopy trees were about 24–30 m tall. On average, oak constituted 50% of the basal area per site (ranging from 14% to 86%), other broadleaved trees 38%, and conifers 12% (for composition of shrub/tree species at the sites, see Table 1). The two *Quercus* species are morphologically similar and were pooled. For the sites, the two plot types (thinning, minimal intervention) did not differ significantly (P > 0.1) in terms of average basal area per hectare or average oak and non-oak tree structure. We have no specific information on tree species composition along the transects where molluscs were sampled (see below). In general, the litter of *Quercus*, and particularly *Picea*, disfavours calcium uptake among the molluscs, while the litter of other broadleaves such as *Fraxinus*, *Populus* and *Corylus* (Table 1) favours molluscs in this respect [27,16].

Our long-term 25 study sites are provided by the following landowners: the state nature conservation units in the county administrations (‘länsstyrelsen’) at Jönköpings län (JL), Östergötlands län (ÖL), Kalmar län (KL) (see www.lst.se) for sites 7 (JL), 11 (ÖL), 12 (ÖL), 13 (ÖL), 15 (ÖL), 20 (KL), 22 (KL), 23 (KL), 24 (KL), 25 (KL); the dioceses (‘stift’) of Linköping and Skara (see www.svenskakyrkan.se/linkopingsstift and www.svenskakyrkan.se/skarastift) for sites 1, 2 (Skara) and 18 (Linköping); the municipalities of Borås (www.boras.se), Växjö (www.vaxjo.se), Oskarshamn (www.oskarshamn.se) for sites 5, 9, 21, respectively; the forest companies Sveaskog (www.sveaskog.se/en/), Boxholms Skogar AB (www.boxskog.se) and Holmen AB (www.holmen.com/en/Forest/) for sites 6, 14 and 17, respectively; the Forest Agency (www.skogsstyrelsen.se/en/) with habitat protection and/or conservation agreement with private forest owners, for sites 3, 4, 10, 19; and two private owners, Dan Ekblad in Järfälla (site 8) and Bo Karlsson in Fröåsa (site 16), that own woodland key habitats. For exact locations of sites, see S1 Fig. All landowners allow collection of specimens (molluscs), either as specified in management plans, or in written agreements. None of the collected mollusc species were threatened, i.e. listed on the Swedish Red List (Swedish Species Information Centre, www.slu.se).

### Experimental treatment and sampling of molluscs

Thinning was conducted from late October 2002 to March 2003. Forest workers cut trees within about 5–8 m from large oaks; cut almost all spruces; cut other broadleaved trees of intermediate size in canopy and sub-canopy; and cut some oaks of intermediate size if they were common. Old, large individuals of other broadleaved trees were usually retained. Although more trees were cut near large oaks, the cut trees were distributed fairly evenly across each plot. For hazel, on average about 50% of all bushes were cut. Cutting was done manually by chain-saw, except at three sites where a harvester was used. At all sites, cut stems were taken from the plots by forwarders, relatively heavy machines that created tracks in the ground where the vegetation was disturbed, and soil often exposed. The length of these tracks was about 300 m per thinning plot. Tops and branches of larger trees, and two oak trunks were left in each plot as deadwood.

On average, 23% (SD = 8.3%, n = 25) of the basal area was cut and harvested in thinning plots. Small understory trees (0.1–4.9 cm in diameter at 1.3 m) were not measured; about 50–90% of them were cut and harvested—a higher proportion if the stem density was high. In Sweden, also smaller trees are of interest for biofuel, and were harvested in our experiment—the project is also supported for testing careful or conservation-oriented biofuel logging. Adding small trees harvested in the understory would increase the proportion of basal area cut to about 26%. This relatively low harvest rate was motivated by the precautionary principle. Canopy openness (% visible sky from the ground) was measured from photographs taken with a digital camera, see [28] for details. Photos were taken in 2001 and again after thinning in late summer 2003. Before thinning in the experimental plot, the mean was 14.1% (SD = 4.3%) and in 2003 was 33.2% (SD = 8.6%, n = 25). This relatively large change was partly caused by the reduction in the understory of trees and bushes, e.g. *Corylus* that may reduce light levels considerably.

Molluscs were sampled using generally recommended methods [29–31]. In the plots we took samples along an 80-m long fixed transect. The transect was placed over central parts of each plot to include important microhabitats for molluscs (ground below the canopies of favourable tree species, stems of large trees, stony ground, dead wood and shady forest floor). Along each transect, we randomly selected 10 squares (each 2.5 x 2.5 m) which were not closer than 1 m to each other (to avoid clustering). In each square, a smaller area (20 x 25 cm) was then randomly selected, and sampled. We removed all litter from each sample area, and sieved the litter in a 8 x 8 mm mesh. The sifted material was taken to the laboratory where it was air-dried. It was then sieved into smaller fractions, and the shells were sorted by hand under a magnifying glass. For each plot (n = 50), all sifted material was pooled. Litter sampling mainly included small snail species. To increase the representation of larger species of snails and slugs in plot samples, and to sample additional habitats, we surveyed tree stems (below about 2 m), dead wood (dead standing trees, logs and stumps), and stones and crevices within 5 m of both sides of each transect. Searching was carried out for a standardized 45-min period and we collected all visible specimens for later identification (if identification was not possible in the field). We refer to this data set as ‘non-litter samples’.

The field sampling before thinning was undertaken over three consecutive autumn seasons (September–October) in 2000 to 2002, covering 8–9 sites per year (one site sampled per day). During these years, the study sites and plots remained undisturbed by forestry operations, and were not subject to extreme weather conditions. Two summer seasons (2003 and 2004) after thinning, in the autumn 2004, we re-sampled all sites using the same methods. Exactly the same squares (2.5 x 2.5 m) along the transects were used in the re-sampling, but the sample area (20 x 25 cm) within the squares was selected randomly. All field sampling was conducted by T.v.P., one of the authors. In 2004, precipitation was on average higher than in 2000–2002 (see below), which might have influenced the results, but control plots (i.e., minimal intervention) make our conclusions strong.

We identified all specimens to species using a magnification of x 6–50, and sorted specimens into age-classes (adults/subadults, >80% of maximum shell size; and juveniles, ≤ 80%). Individuals already dead before the sampling (shells) was a separate class, excluded from the analyses since differences in ground chemistry lead to differential decomposition of shells: in acid sites they disappear in a few months, while in sites on calcareous ground they may be preserved for decades. The nomenclature of molluscs follows Falkner et al. [32]. The mollusc species recorded were classified in two groups, by main habitat type or preference in southern Sweden: 1) forest species, and 2) open habitat/generalist species (see Table with species below). Our classification may differ from classifications in other parts of the species’ distributions; for details regarding the classification, see [15] and Table 1 therein.

### Calculations and statistical analyses

To test changes in species richness, we compare the two plot types before versus after treatment (thinning). The test variable was number of species recorded after, minus before treatment, and we used paired t-tests since the plots (n = 25+25) were geographically paired and on average similar in forest composition before thinning. Thus, a negative value indicates a decrease in species richness. Because we predicted a general decline in species richness due to treatment, we used a one-tailed test. All age classes of molluscs were pooled. Since slugs might respond differently from snails, we also tested the data with only snails included (which provided the largest sample size). Moreover, we tested forest species separately, since we expected that they would respond stronger to thinning than all species combined.

To examine changes in individual abundance of molluscs, species and age classes were first pooled for an overall test. The test variable was number of individuals recorded after, minus before thinning in a plot (paired t-test). A negative value indicates decrease, a positive value increase of individuals. We used a one-tailed test, as the abundance of molluscs was predicted to be negatively affected by thinning. For tests of abundance in individual species we used two-tailed tests, as we did not predict these responses. When a species had not been found before or after the thinning (a zero value) on either the thinning or the minimal intervention plot, the site was excluded from the analyses. If a zero indicated no change in the amount of individuals (e.g. 10 individuals before and 10 individuals after thinning), a plot was included in the analyses. For meaningful statistical tests, we required that a species must have been present on four or more sites (thus, n ≥ 4). For the remaining species, we only present descriptive statistics. For individual species, we set p ≤ 0.01 as the level for statistical significance, since many tests were done.

The species were assigned ‘insufficient sample size’ or a reaction (positive, negative, neutral) based on the mean change in number of individuals due to thinning, compared to the mean change in number of individuals on minimal intervention plots. We used a one sample chi-squared test to determine if the number of positive and negative reactions (species) deviated from random.

For analysis of effects on the reproduction of snails, we used litter samples only (some non-litter samples lacked age class determinations). Due to small sample sizes, we only included species and plots where we had collected at least 5 adult individuals. From those plots, all juveniles of the species were summed, as were adults/subadults. The overall percentage of juveniles (not mean value) among all specimens was calculated, based on 19 thinning plots and 19 reference plots (with at least one species that met the requirements), and then compared before and after thinning. We used the same plots in another analysis, testing the difference between the change in percentage juveniles on minimal intervention plots and the change in percentage juveniles on thinning plots, and the difference between before and after for both thinning and minimal intervention plots (two-tailed paired t-test). For individual species, we analysed those which occurred in sufficient number (5 adults) on at least 3 sites, and where individuals occurred on both thinning and minimal intervention plots before and after thinning. Six species qualified for this analysis, but they occurred on too few sites for meaningful paired t-tests, so we only present descriptive statistics for them.

We analysed if there was any change in general species composition in the conservation thinning plots compared to the minimal intervention plots by running Adonis in the vegan package in the program R3.0.1 [33]. Adonis is a multivariate analysis of variance based on dissimilarity measures, typically used for testing if multidimensional groups differ from each other. As response variable we used the full matrix of abundances before and after in the two plots of each site. As explanatory variables we included treatment (treatment versus minimal intervention plot), time (before or after the intervention), and their interaction. We also constrained the analyses by including the blocking factor site. As distance measure we used Bray-Curtis dissimilarity index, which also is default in Adonis. A significant interaction between treatment and time would suggest an effect of the thinning on the species composition of the molluscs. In a second analysis, we analysed presence-absence data (0–1) for species in the same way.

To illustrate the pattern in species composition among treatment and minimal intervention plots and times we plotted the scores from an NMDS-ordination with two axes analysed in the vegan package of R. As input data we used the same species-by-plot matrix as in the Adonis analysis. We also tested if a three-dimensional ordination would capture the gradients in species composition better than a two-dimensional.

## Results

### Species richness and species abundance

We identified in total 53 species; 41 snails and 12 slugs, ranging from 7 to 29 species in thinning and 8 to 30 in minimal intervention plots, with means (treatment/time) ranging from 16.4 to 19.2 (Table 2). The three most common snail species were *Nesovitrea hammonis*, *Euconulus fulvus*, and *Punctum pygmaeum*; the most common slug was *Arion fuscus* (see also below).

**Table 2:** Descriptive statistics for the number of species found on all sites (n = 25).

Treatment	Mean no. of species	S. E.	Minimum	Maximum
Thinning plot before	17.8	1.0	7	29
Thinning plot after	16.4	1.0	8	25
Minimal interv. plot before	18.6	1.0	8	27
Minimal interv. plot after	19.2	1.2	10	30

**Fig 2:**
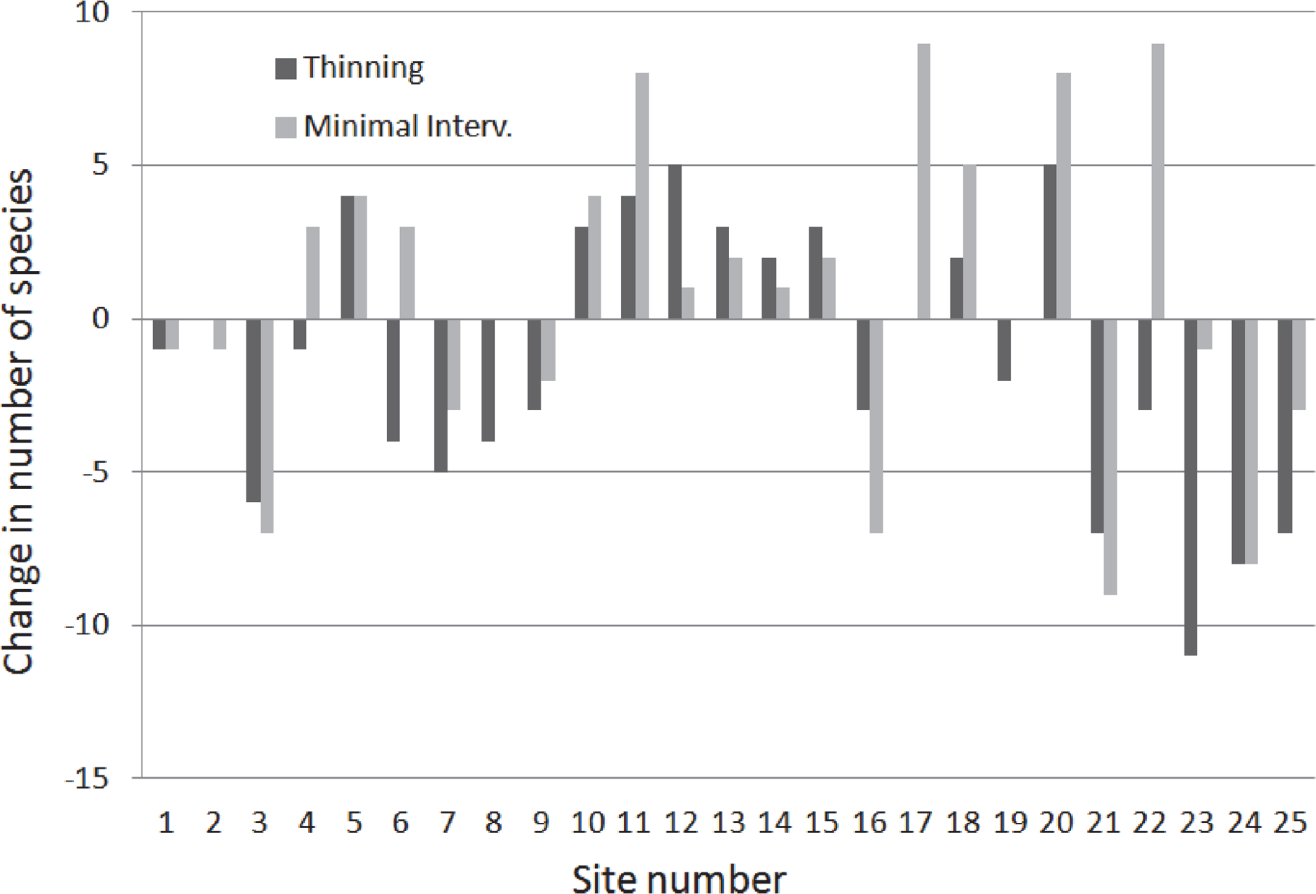
Changes in species richness (number of recorded species; after minus before treatment) of snails and slugs, for thinning plots and minimal intervention plots. For site number and location, see Fig. 1.

We found a mean change of minus 1.4 ± 0.9 species (mean ± SE, n = 25) of snails and slugs on thinning plots, while minimal intervention plots had a mean change of plus 0.7 ± 1.0 species (n = 25) between the two inventories. We found much variation among sites in these changes (see Fig. 2) but the difference between thinning and reference plots was significant (t = -2.5, one-tailed p = 0.010). When slugs were excluded from the data sets, the mean change for the thinning plots was minus 0.8 ± 0.9 species, and the corresponding value for minimal intervention plots plus 1.0 ± 1.0 species, also a significant though weaker difference between plot types (t = -2.1, one-tailed p = 0.024).

The same analysis was done for forest species, i.e. with 12 species classified as generalists excluded (Table 3). The change for forest species on thinning plots was minus 0.2 ± 0.7 species and on minimal intervention plots plus 1.2 ± 0.1 species (t = -2.1, one-tailed p = 0.022).

**Table 3:** Mean change in abundance and results of paired t-tests for each species; *indicates non-forest-species.

Species	Mean change ± SE		n	t	p	Trend
	Thinning	Minimal intervention				
**Snails**						
*Acanthinula aculeata*	25.4 ± 10.3	48.7 ± 21.3	7	-1.666	0.147	↓
*Aegopinella nitidula*	-2.3 ± 5.3	8.7 ± 4.2	3			(↓)
*Aegopinella pura*	-1.2 ± 1.7	3.6 ± 3.6	13	-1.277	0.226	↓
Arianta arbustorum*	-2.0 ± 1.9	0.00 ± 2.02	20	-1.215	0.243	↓
*Carychium minimum*	-7.5 ± 6.3	-24.2 ± 25.8	4			(↑)
*Carychium tridentatum*	14.2 ± 9.0	50.5 ± 32.2	12	-1.057	0.313	↓
*Cepaea hortensis*	3.8 ± 3.3	2.6 ± 1.3	19	0.415	0.683	↑
*Clausilia bidentata*	-0.6 ± 0.7	3.2 ± 1.3	17	-3.233	0.005	↓
*Clausilia pumila*	6.0	30.0	1			(↓)
Cochlicopa lubrica*	0.9 ± 6.4	9.2 ± 6.2	18	-1.222	0.238	↓
*Cochlicopa lubricella*	5.3 ± 3.4	11.4 ± 4.7	15	-1.051	0.311	↓
*Cochlodina laminata*	2.1 ± 1.9	11.6 ± 2.5	16	-3.770	0.002	↓
*Columella aspera*	1.1 ± 2.4	4.7 ± 2.6	16	-2.245	0.040	↓
*Columella edentula*	17.3 ± 10.0	11.5 ± 6.1	8	0.687	0.514	↑
*Discus rotundatus*	0.3 ± 2.2	-4.2 ± 10.8	6	0.381	0.719	↑
*Discus ruderatus*	-0.7 ± 0.9	1.8 ± 2.3	9	-0.935	0.377	↓
Euconulus fulvus*	3.4 ± 6.5	22.9 ± 8.4	23	-3.522	0.002	↓
*Euconulus trochiformis*	/	-9.0	1			/
*Euomphalia strigella*	-7.6 ± 9.63 (n = 5)	2.3 ± 2.6	4			(↓)
*Fruticicola fruticum*	3.3 ± 3.0	-3.5 ± 8.4	4	0.952	0.411	↑
*Helicigona lapicida*	-1.6 ± 1.0	1.2 ± 1.6	10	-1.332	0.216	↓
*Macrogastra plicatula*	-1.0 ± 1.6	-2.6 ± 2.9	5	0.544	0.616	↑
*Merdigera obscura*	-1.5 ± 0.5	0.7 ± 0.9 (n = 3)	2			(↓)
*Monachoides incarnatus*	/	-1.0	1			/
Nesovitrea hammonis*	12.4 ± 7.7	18.2 ± 5.3	25	-1.153	0.260	↓
*Nesovitrea petronella*	2.9 ± 1.9	12.3 ± 6.4	11	-1.543	0.154	↓
*Oxychilus alliarius*	-2.4 ± 0.8	-1.7 ± 1.4	7	-0.884	0.411	↓
Oxychilus cellarius*	5.3 ± 7.9 (n = 3)	-33.0 ± 30.0	2			(↑)
*Punctum pygmaeum*	67.4 ± 27.6	136.4 ± 35.4	23	-2.961	0.007	↓
*Succinea putris*	-2.3 ± 2.0 (n = 4)	-2.0 ± 1.5	3			(↓)
Trichia hispida*	-1.0 ± 0.6	-1.4 ± 1.6	5			(↑)
*Vallonia costata*	-58.5 ± 57.5	1.0 ± 3.6 (n = 3)	2			(↓)
*Vallonia excentrica*	/	-1.0	1			/
*Vertigo alpestris*	/	-0.3 ± 0.7	3			/
*Vertigo pusilla*	7.9 ± 3.5	21.4 ± 9.5	8	-1.999	0.086	↓
*Vertigo ronnebyensis*	/	1.0	1			/
*Vertigo substriata*	20.4 ± 9.4	47.7 ± 14.5	18	-2.916	0.010	↓
*Vitrea contracta*	0.8 ± 0.8	2.1 ± 1.1	9	-1.940	0.088	↓
*Vitrea crystallina*	-2.2 ± 1.7	-2.2 ± 4.1	5	0.000	1.000	↕
Vitrina pellucida*	-3.0 ± 2.0	-0.5 ± 1.7	13	-1.004	0.335	↓
*Zonitoides nitidus*	-1.0	-1.5 ± 8.5 (n = 2)	1			(↑)
**Slugs**						
*Arion ater*	0.3 ± 0.3	0.7 ± 0.6	7	-1.162	0.289	↓
Arion circumscriptus*	1.8 ± 1.3 (n = 4)	3.7 ± 3.2	3			(↓)
Arion fasciatus*	-0.4 ± 1.1	1.9 ± 0.9	12	-1.728	0.112	↓
*Arion fuscus*	0.4 ± 0.2	0.7 ± 0.3	25	-0.781	0.442	↓
*Arion intermedius*	/	0.5 ± 0.5	2			/
Arion silvaticus*	0.0 ± 0.2	-0.1 ± 0.3	14	0.249	0.807	↕
Arion vulgaris*	34.0	13.0	1			(↑)
*Deroceras laeve*	-0.5 ± 0.5 (n = 2)	1.0	1			(↓)
Deroceras reticulatum*	4.0	4.5 ± 2.5 (n = 2)	1			(↓)
*Lehmannia marginata*	-0.4 ± 0.3	-0.3 ± 0.5	20	-0.256	0.800	↕
*Limax cinereoniger*	-0.3 ± 0.5	0.0 ± 0.4	4	-0.522	0.638	↓
*Malacolimax tenellus*	0.4 ± 0.3	0.4 ± 0.5	23	-0.110	0.913	↕

Trend is change in thinning in comparison to minimal intervention plots (weak trend in parentheses).

**Fig 3:**
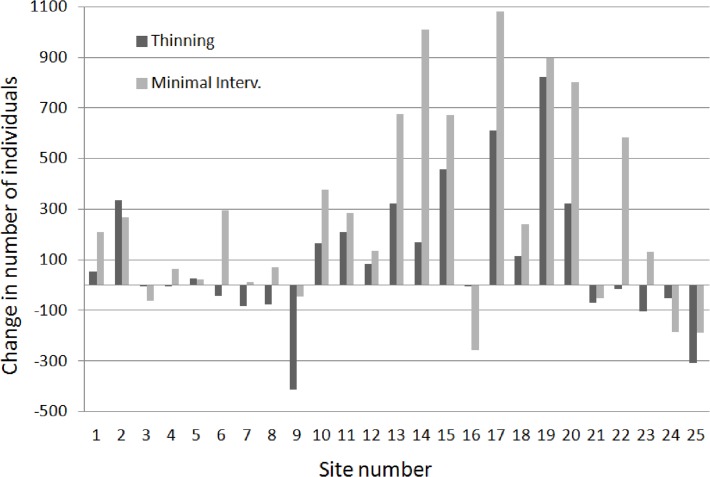
Changes in abundance (total number of individuals; after minus before treatment) of snails and slugs, for thinning plots and minimal intervention plots. For site number and location, see Fig. 1.

In total, 24 369 land molluscs were alive at the time of sampling. Before the thinning, we sampled 3682 specimens on the minimal intervention plots and 3 667 on the thinning plots. After the treatment, 10 802 specimens were found on the minimal intervention plots and 6218 on the thinning plots. The mean change (after-before) in total abundance was plus 101 ± 54.0 on thinning, and plus 281 ± 77.3 on minimal intervention plots (t = -3.7, one-tailed p < 0.001). Thus, thinning had a clear negative effect, but the number of individuals increased on almost all plots, with much variation (Fig. 3).

All 53 species recorded are listed in Table 3, with mean ± SE change in number of individuals (after-before), and number of sites included. For 29 species, a reaction was assigned, whereas data were insufficient, or no reaction/trend was visible, in 24 species. Of the 29 species with reaction, 24 showed a negative, and five species a positive reaction, refuting the null hypothesis of random species response (p < 0.001, chi-square test). We found strong negative reactions for six species: *Clausilia bidentata*, *Cochlodina laminata*, *Columella aspera*, *Euconulus fulvus*, *Punctum pygmaeum* and *Vertigo substriata* (Table 3). No species with positive reaction had a significant response.

### Reproduction

On minimal intervention plots the overall percentage (not mean value) of juveniles based on *4*9 sites was 52% before and 77% after treatment, whereas on thinning plots, 48% juveniles were found before, and 74% after thinning (Table 4).

**Table 4:** Descriptive statistics for the proportion of juveniles, summed over all species (with sufficient sample size), for n = 19 sites with sufficient number of specimens to calculate the proportions.

Treatment	Mean proportion	S. E.	Minimum	Maximum
Thinning plot before	0.39	0.05	0	0.88
Thinning plot after	0.54	0.06	0	0.90
Minimal interv. plot before	0.45	0.05	0.10	0.82
Minimal interv. plot after	0.66	0.05	0.16	0.90

For criteria in this analysis, see text.

The mean change in the percentage juveniles over all species on thinning plots (before-after) was plus 14.5 ± 7.4 percent units (n = 19 sites), and on minimal intervention plots plus 21.5 ± 7.7 percent units (n = 19 sites), a difference which was not significant (t = -1.1; two-tailed p = 0.30). Testing plot types separately, we found that the percentage juveniles increased on minimal intervention plots from 2000/01/02 to 2004 (t = -2.8; two-tailed p = 0.012). It increased also on thinning plots, but not significantly so (t = -1.0; two-tailed p = 0.067).

**Fig 4:**
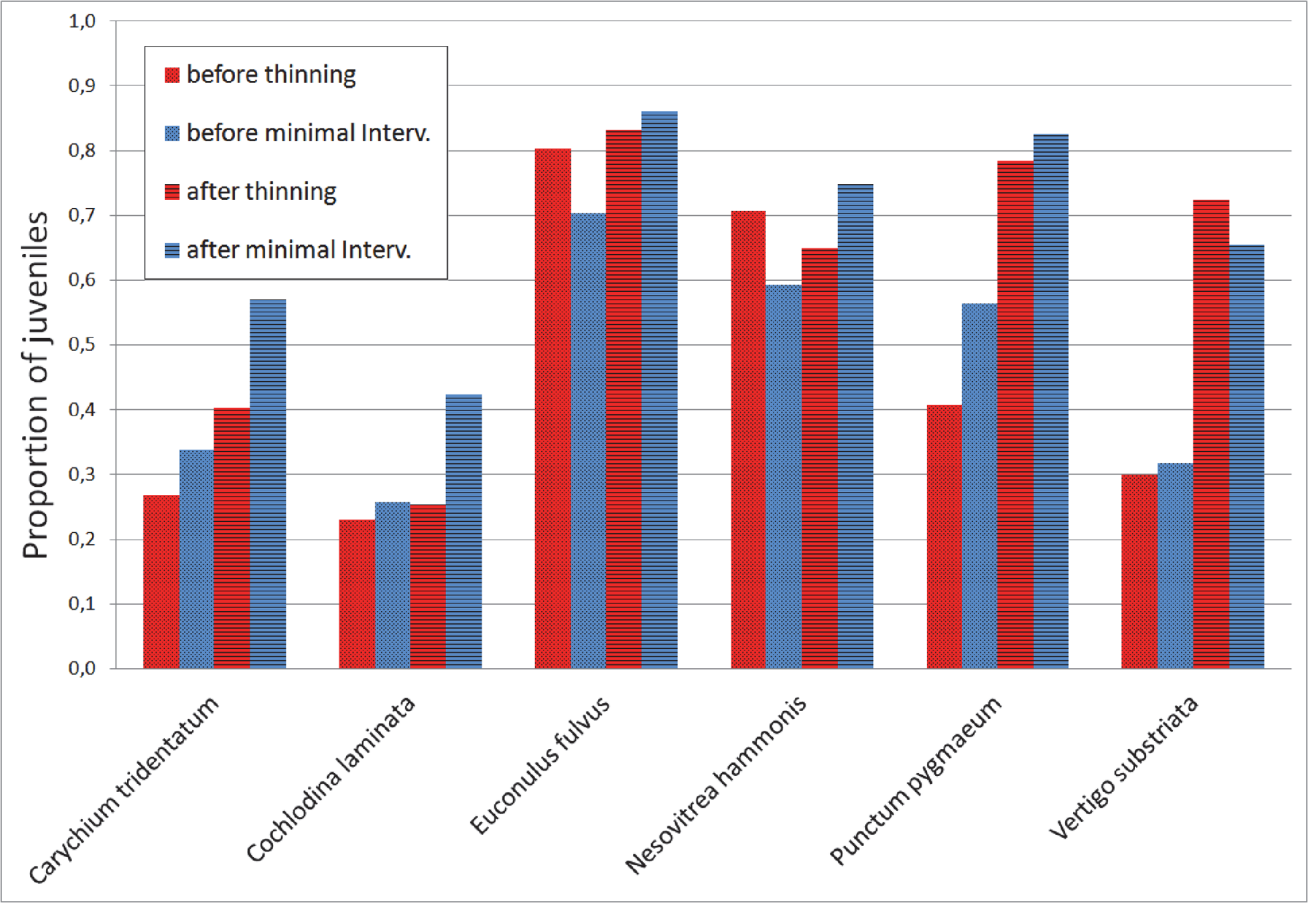
The overall proportion of juveniles for six species with sufficient samples. Data for thinning plots (pooled) and for minimal intervention plots (pooled; for details, see text).

The overall proportion of juveniles for six species that met the requirements for analysis indicates negative response in reproduction after thinning (compared to minimal intervention) in *Cochlodina laminata*, possibly also in *Carychium tridentatum*, while no clear pattern was evident in the other four species (Fig. 4). The result for *Punctum pygmaeum* should be treated with caution, since some or many individuals may reach maturity in the smaller size classes (T.v.P, pers. obs.).

### Species communities

There was no significant change in species composition caused by the thinning (p = 0.28 for the interaction between time [before vs. after] and treatment [thinning vs. minimal intervention plots]; see S1 Table, for model output). No qualitative differences were found if only presence/absence data were analysed (results not shown).

**Fig 5:**
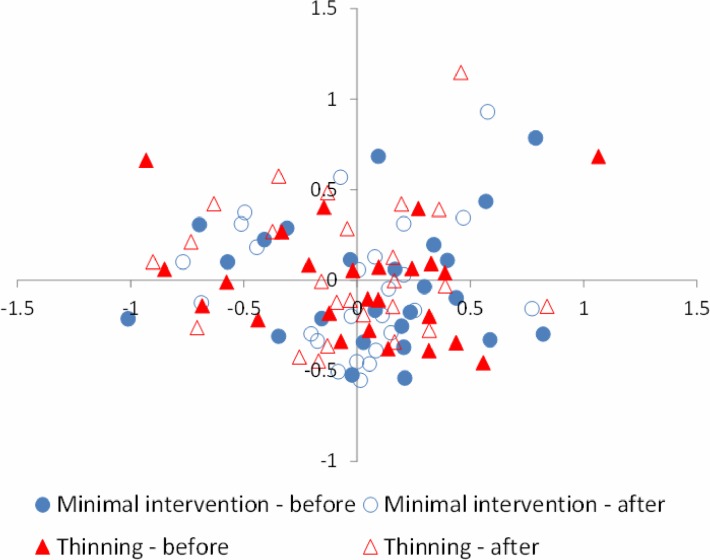
Variation in species composition between the plots: before-after in treatment plots (thinning) and before-after in minimal intervention plots (reference). Analysis based on a two dimensional ordination, using abundance data (NMDS, stress-value 22).

The large overlap in species composition between years and between treatment and minimal intervention plots is also seen in an NMDS-ordination plot (Fig. 5). No qualitative differences were seen if only presence/absence data were analysed or if three instead of two axes were used in the NMDS-analysis (results not shown).

## Discussion

### Species richness and abundance

The predicted negative effect of conservation thinning of closed-canopy mixed oak forest on land molluscs was confirmed: using a strong BACI experimental design including many replicates at landscape level, we show that in the short-term thinning reduces species richness, reduces overall abundance of molluscs, and affects certain mollusc species negatively. However, the effect should be interpreted as mild, and probably transient (see below).

In 2004, the overall abundance of snails had increased about threefold on minimal intervention plots and twofold on thinning plots. Precipitation, but no other factor of known major importance, changed for both plot types over the sampling period. SMHI data (www.smhi.se) for seven sites in the region (Borås, Ulricehamn, Norrköping, Gladhammar, Målilla, Växjö, Öland) reveal that the mean summer precipitation was highest in 2004 (for June-September in 2000, 2001, 2002, 2003 and 2004 the mean values are 262.5, 327.3, 218.3, 305.3, and 341.3 mm, respectively). Wet conditions may lead to a marked increase in mollusc populations [34,35]. Food availability and egg and offspring survival may increase, which would explain the high proportion of juveniles in the autumn 2004 in both thinning and minimal intervention plots. In addition, the amount of rainfall normally decreases from west to east in southern Sweden and is often geographically patchy in summer, which probably contributes to the variation in mollusc responses seen among our sites.

While the increases in abundance and species richness were significantly higher in minimal intervention than in thinning plots, it is important to note that even though the abundance of molluscs increased also on thinning plots, the species richness declined there. Thus, a negative effect of conservation thinning is clear; with a higher abundance, the sampling would normally be expected to lead to more species. This was seen under minimal intervention, where both abundance and species richness increased.

We suggest that factors directly associated with the conservation thinning (e.g. ground disturbances, increased canopy openness, increased ground temperature, reduced stem numbers, reduced litter) caused the negative effects on the mollusc fauna. In our landscape study [15] local pH of litter, linked also to calcium availability, was important in explaining variation in species richness and variation in species composition among the sites. However, in thinning plots, pH of litter before versus after thinning did not differ (unpubl. data), so factors such as increased openness and reduced amounts of litter may be more important.

We consider the effect of thinning as mild and suggest that habitat heterogeneity, including undisturbed parts in thinning plots, and refuges for molluscs in hollows and crevices, rocks, and dead wood (logs, snags or stumps) reduced the negative effect of increased openness, see [22,36]. Variation in hollows/crevices in the ground at sites contributed as explanatory variable to variation in species richness among the sites [15].

### Responses in the species

The species richness analysis where we used all mollusc species yielded the lowest p-value, which we judge to be an effect of sample size (more species included). Sample sizes for slugs (species, individuals) were much smaller than for snails. Six or seven of the 13 slug species tended to react negatively, and only one tended to react positively. Our results for slugs should be interpreted with caution; they are more mobile than snails and more sensitive to weather (especially drought), and there is a higher probability that they are overlooked during sampling, [35] and T.v.P (pers. obs.).

Surprisingly, to judge from the quantitative changes in the plots, the impact of thinning on species richness of forest species was weaker than was the case for analyses of all species combined, or all species except slugs. Almost all generalist species with sufficient samples reacted negatively. The forest species were classified subjectively [15] by T.v.P., on the basis of much experience and many earlier studies in Sweden. However, among the six species with the most negative reaction to thinning, five were classified as forest species (*Clausilia bidentata*, *Cochlodina laminata*, *Columella aspera*, *Punctum pygmaeum* and *Vertigo substriata*). These species had smaller increases or even decreased (*C*. *bidentata*) on the thinning plots after treatment (though *V*. *substriata* increased much in absolute numbers). The percentage juveniles of *C*. *laminata* clearly increased on minimal intervention plots, but not on thinning plots. *C*. *laminata* prefers shaded woods and often climbs on tree trunks [34]; since many trunks, and a high proportion of the smaller trunks, were removed by thinning, its habitat was partly reduced. Moreover, at several sites we removed stems of e.g. *Fraxinus*, *Populus*, and *Corydalis* that create favourable litter for molluscs [27], which in turn may have disfavoured *C*. *laminata* (and other molluscs).

### Reproduction and community change

The total number of individuals in 2004 was much higher on minimal intervention than on thinning plots but the overall increase in the proportion of juveniles did not differ between plot types, and we did not find any indication of reduced reproduction in thinning plots. This suggests that reproduction was little affected by conservation thinning, and that thinning rather reduced the total number of individuals. Thus, adults and juveniles seem to be affected in much the same way.

The ordination analyses indicated that overall community composition did not change due to conservation thinning. The likely reason for these results is that thinning essentially affects all species similarly; and the stronger negative effect on a few species were not strong enough to influence the overall community composition. Comparing undisturbed boreal forest with clear-cuts, Hylander [16] found no significant difference in species composition of snails, which is consistent with our results.

Our experimental study should be considered as short-term. Increased species richness in forbs and grasses due to thinning was quantified already in July-August 2003 [37] and may have contributed to the mild thinning effect on molluscs. After 2003 the herbs in the field layer continued to increase, and deciduous broadleaved shrubs (mainly *Corylus avellana*, *Frangula alnus*) and small trees increased even more [see photographs, pdf ‘Nyhetsbrev 5’, pp. 12–17, www.bioenv.gu.se/personal/Gotmark_Frank/]. This should lead to more cover and food for molluscs, and we predict that the molluscs are favoured in the long term at most of the sites. This prediction remains to be tested, however. For snails in moist stream-side boreal forest [23] there is evidence for long-term positive effects after clear-cutting, due to regeneration of broadleaved trees, see also [35].

### Conclusions and implications for temperate conservation forests

Conservation thinning reduces the species richness and abundance of land molluscs in closed-canopy mixed oak forests with conservation values. Minimal intervention is probably a better option in habitat management for land molluscs in this type of forest, as long as conifers do not increase, and taxa such as *Corylus*, *Fraxinus*, and *Populus* that produce litter with extractable calcium are available. But in the short term at least, conservation thinning for oak survival and oak regeneration favours vascular plants, lichens and beetles (for our results regarding all organism groups, see [6] and Table 3 and Example Section 3 therein). We tentatively judge that the negative effects on fungi [7] and molluscs (present study) are transient. So far, conservation thinning can be recommended. However, it should be combined with minimal intervention to create heterogeneity in forest habitats, including occurrence of old trees and dead wood [38,39]. In addition, as far as possible, active management and minimal intervention should be combined with long-term research—crucial for firm conclusions about habitat management of conservation forests [21,6].

## Supporting Information

S1 Dataset Supporting data. (XLS)

S1 Fig Coordinates and map of study sites. (DOCX)

S1 Table Model outcome from the Adonis-analysis of the species composition taking the blocked design into account. (DOC)
