# Supplementary material for: Conservation Thinning in Secondary Forest: Negative but Mild Effect on Land Molluscs in Closed-Canopy Mixed Oak Forest in Sweden
Source: PLoS One. 2015 Mar 24;10(3):e0120085. doi: 10.1371/journal.pone.0120085 (PMC4372413; doi:10.1371/journal.pone.0120085)
Supplement: S1 Fig — (DOCX) [file pone.0120085.s002.docx]

**Supplementary material.**

Model outcome from the Adonis-analysis of the species composition taking the blocked design into account.

__________________________________________________________________________

Df Sums of sqs Mean sqs F.Model R2 Pr(> F)

Before/After 1 1.2837 1.28365 4.8882 0.04762 0.001 ***

Reference/treatment 1 0.2795 0.27951 1.0644 0.01037 0.061 .

Interaction 1 0.1820 0.18200 0.6931 0.00675 0.279

Residuals 96 25.2100 0.26260 0.93526

Total 99 26.9551 1.00000

__________________________________________________________________________
