# Supplementary material for: Conservation Thinning in Secondary Forest: Negative but Mild Effect on Land Molluscs in Closed-Canopy Mixed Oak Forest in Sweden
Source: PLoS One. 2015 Mar 24;10(3):e0120085. doi: 10.1371/journal.pone.0120085 (PMC4372413; doi:10.1371/journal.pone.0120085)

**Supplementary material.** Location of study sites in the Swedish Oak Project (coordinates and map)

| **Nr.** | **Site name)** | **Län** **(County)** | **X-koordinat** | **Y-koordinat** |
| --- | --- | --- | --- | --- |
|  |  |  |  |  |
| 1 | Skölvene NB | Västra Götalands län | 6435900 | 1342710 |
| 2 | Karla NB | ” | 6448228 | 1333240 |
| 3 | Östadkulle BS | ” | 6438527 | 1312401 |
| 4 | Sandviksås NA | ” | 6422456 | 1325557 |
| 5 | Rya åsar NR | ” | 6404716 | 1328335 |
| 6 | Strakaskogen NB | ” | 6451127 | 1397122 |
| 7 | Bondbergets NR | Jönköpings län | 6406533 | 1406729 |
| 8 | Långhults NB | Kronobergs län | 6299701 | 1405344 |
| 9 | Bokhultets NR | ” | 6304778 | 1437963 |
| 10 | Kråksjö by NB | ” | 6279102 | 1472274 |
| 11 | Stafsäter NR | Östergötlands län | 6463954 | 1492277 |
| 12 | Åtvidaberg NB | ” | 6451645 | 1513420 |
| 13 | Fagerhults NB | ” | 6437257 | 1483099 |
| 14 | Aspenäs NB | ” | 6430484 | 1470012 |
| 15 | Norra Vi NB | ” | 6416860 | 1471798 |
| 16 | Fröåsa NB | ” | 6418101 | 1491305 |
| 17 | Ulvsdal NB | Kalmar län | 6437347 | 1524191 |
| 18 | Hallingeberg NB | ” | 6412083 | 1526971 |
| 19 | Ytterhult NB | ” | 6396388 | 1548643 |
| 20 | Fårbo NB | ” | 6365604 | 1538890 |
| 21 | Emsfors NB | ” | 6336257 | 1539723 |
| 22 | Getebro NR | ” | 6321599 | 1521890 |
| 23 | Lindö NR | ” | 6296250 | 1539339 |
| 24 | Lilla Vickleby NR | ” | 6271764 | 1539205 |
| 25 | Albrunna NR | ” | 6243586 | 1537705 |

# Map, see next page (2)

(Border are counties;usually indicated on Swedish maps)

# Southern Sweden


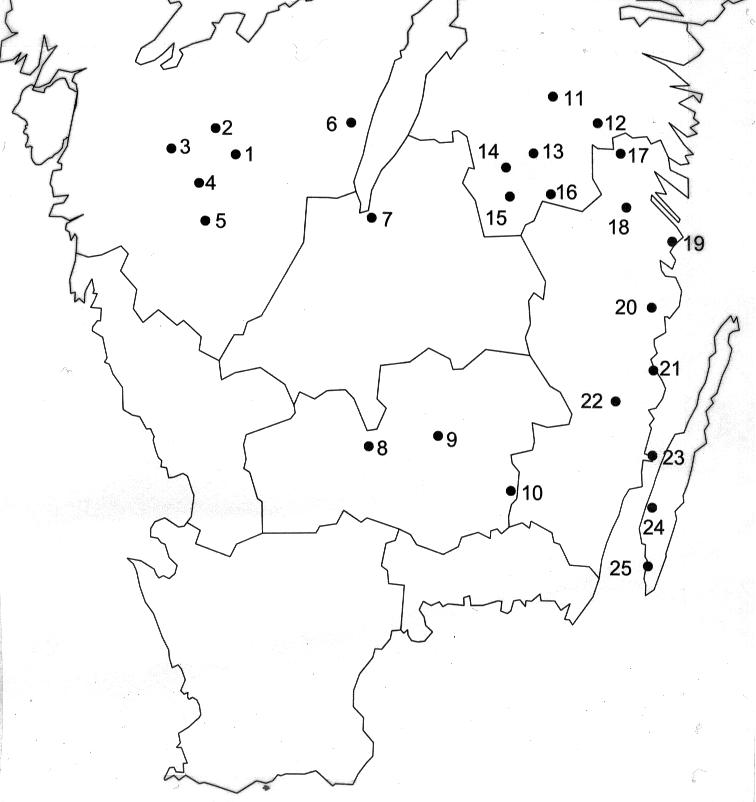

Supplement: S1 Table — (DOC) [file pone.0120085.s003.doc]
